# Shikonin attenuates rheumatoid arthritis by targeting SOCS1/JAK/STAT signaling pathway of fibroblast like synoviocytes

**DOI:** 10.1186/s13020-021-00510-6

**Published:** 2021-10-02

**Authors:** Lianhua He, Huijie Luan, Juan He, Miaomiao Zhang, Qingxia Qin, Yiping Hu, Yueming Cai, Desheng Sun, Yu Shi, Qingwen Wang

**Affiliations:** 1 Department of Rheumatism and Immunology, Peking University Shenzhen Hospital, Shenzhen Peking University, The Hong Kong University of Science and Technology Medical Center, No.1120, Lianhua Road, Futian District, Shenzhen, 518036 China; 2 The Key Laboratory of Inflammatory and Immunology Diseases, Shenzhen, China; 3 Department of Ultrasound, Peking University Shenzhen Hospital, Shenzhen, China

**Keywords:** Shikonin, Collagen-induced arthritis, suppressor of cytokine signaling 1, Fibroblast-like synovial cells

## Abstract

**Background:**

Rheumatoid arthritis is a progressive and systemic autoimmune disease seriously compromises human health. Fibroblast like synoviocytes are the major effectors of proliferation and inflammation in rheumatoid arthritis synovial tissue. Shikonin has anti-inflammatory and immunomodulatory activities. But, its role on synovitis of rheumatoid arthritis is unknown.

**Methods:**

The DBA/1 male mice were randomly divided into the following three groups (n = 6): (1) the normal control group of mice, (2) the CIA (collagen-induced arthritis) group in which mice suffered from arthritis induced by collagen, (3) the SKN (shikonin) group of mice which got arthritis and given intragastrically with shikonin 4 mg/kg per day continuously for 20 days,(4) the MTX (methotrexate) group of mice which got arthritis and orally administration with shikonin 0.5  mg/kg once two days continuously for 20 days. The therapeutic effect of shikonin on collagen induced arthritis mice was tested by arthritis incidence rate, arthritis score and inflammatory joint histopathology. The invasion, adhesion and migration of fibroblast like synoviocytes induced by tumor necrosis factor-α were applied to measure the anti-synovitis role of shikonin. The effect of shikonin on expression of interleukin-6, interleukin-1β and tumor necrosis factor-α was measured by enzyme linked immunosorbent assay. The interaction between shikonin and suppressor of cytokine signaling 1 was verified by molecular docking. The signaling pathways activated by shikonin were measured by western blot.

**Results:**

Shikonin decreased the arthritis score and arthritis incidence, and inhibited inflammation of inflamed joints in collagen induced arthritis mice. And shikonin reduced the number of vimentin^+^cells in collagen induced arthritis mice inflamed joints. Meanwhile, shikonin suppressed tumor necrosis factor-α-induced invasion, adhesion and migration of fibroblast like synoviocytes and reduced the expression of interleukin-6, interleukin-1β and tumor necrosis factor-α. And we found that shikonin targeted suppressor of cytokine signaling 1. More interestingly, shikonin blocked the phosphorylation of Janus kinase 1/signal transducer andactivator of transcription 1/signal transducer andactivator of transcription 6 in synovial tissues and in fibroblast like synoviocytes.

**Conclusion:**

Shikonin represents a promising new anti-rheumatoid arthritis drug candidate that has anti-synovitis effect in collagen induced arthritis mice and inhibits tumor necrosis factor-α-induced fibroblast like synoviocytes by targeting suppressor of cytokine signaling 1/ Janus kinase/signal transducer andactivator of transcription signaling pathway. These findings demonstrate that shikonin has anti-synovitis effect and has great potential to be a new drug for the treatment of rheumatoid arthritis.

**Supplementary Information:**

The online version contains supplementary material available at 10.1186/s13020-021-00510-6.

## Introduction

Rheumatoid arthritis (RA) is a systemic, progressive and autoimmune disease, which characterized by chronic inflammatory synovial hyperplasia, hypertrophy and bone joint invasion, which seriously compromises human health [1–3]. Fibroblast like synoviocytes (FLS) are the major effectors of proliferation and inflammation in RA synovial tissue, which have the biological characteristics of abnormal proliferation, migration, adhesion, invasion and secretion, playing a major role in the pathology of RA [4]. Therefore, inhibiting the abnormal function of synovial cells in RA can effectively control synovial inflammation and proliferation, prevent the erosion of bone and cartilage, and finally attenuate the progression of RA.

Suppressor of cytokine signaling (SOCS) plays an important role in the immune system, which is a class of protein transduction molecules [5]. By now, eight members of SOCS protein family have been found, containing CIS and SOCS1-7, among which SOCS1 is the most important member [6]. Janus kinase/signal transducer andactivator of transcription (JAK/STAT) is involved in the regulation of a variety of pathophysiological processes including cell proliferation, immune regulation, and inflammatory response, which is an important multi-functional cytokine transduction pathway [7]. In the synovium of osteoarthritis patients, JAK and STAT1 were rarely expressed, while in the synovium of RA patients were highly expressed [8]. SOCS1 is a negative regulator of JAK/STAT, which plays an important role in inflammation, autoimmune diseases and other diseases [9].

Shikonin (SKN) is the crucial active chemical component purified from dried roots of traditional Chinese medicine *arnebia euchroma* (zicao) [10, 11]. SKN has excellent biological activity including anti-inflammation, immunosuppression, abirritation, and so on. It was reported that SKN inhibits the progress of collagen induced arthritis (CIA) in mice [12, 13], which could relieve the swelling and cartilage destruction of joint in CIA mice [12, 14]. In addition, SKN inhibits pro-inflammatory cytokines including interleukin -6 (IL-6), tumor necrosis factor-α (TNF-α), interleukin -12 (IL-12), interleukin -8 (IL-8), and interleukin -17A (IL-17A), and simultaneously induced anti-inflammatory interleukin-10 (IL-10), mediators interleukin-4 (IL-4) and transforming growth factor-β (TGF-β) in CIA mice and in anti-collagen monoclonal antibody-induced arthritis in mice as well as FLS in vitro [12–17]. However, the specific action targets and underlying mechanism of SKN on treating RA remains unclear. In our study, we sought to explore the specific action targets and underlying mechanism of SKN on treating RA and found that SKN got the effect of anti-synovitis in CIA mice and inhibits TNF-α-induced FLS by targeting SOCS1 and the signaling pathway of SOCS1/JAK/STAT. Our data would provide foundation for the application of SKN in anti-RA area.

Since the 1950s, methotrexate (MTX) has been used for the treatment of RA and provided good results. Although there are many new drugs at present, MTX is still widely used for the treatment of RA and is often used as a positive control in animal experiments. Therefore, MTX was selected as a positive control drug in our animal experiments.

## Materials and methods

### Reagents, media, and antibodies

The powder of SKN (purity > 98%) and MTX were purchased from Shanghai Yuanye Biotechnology Co., Ltd. Fetal bovine serum and H-DMEM were obtained from GIBCO; Tetrazolium bromide reduction (MTT) was purchased from Sigma. Protease inhibitor, mixed lysis buffer, protein phosphatase inhibitors, and hypersensitive luminescent solution were achived from Beijing Puli Lai Gene Technology Co. Ltd. SDS-PAGE protein buffer (5 ×) and Bradford protein concentration assay kit from Biyun Tian biotechnology; Matrigel was obtained from Corning; Transwell chambers and recombinant human TNF-α from Costar and PeproTech respectively; horseradish enzyme-labelled goat anti-rabbit antibody and adhesion protein from Millipore; anti-fluorescence quenching coating agent from Southern Biotech; Dimethyl sulfoxide (DMSO) was purchased from Amresco; SDS, crystal violet, glycine and Tris alkali powder were purchased from the meimen Lun Biotechnology Co. Ltd.; DAPI was purchased from AAT Bioquest company; complete and incomplete Freund’s adjuvant (C/IFA), bovine type II collagen were got from Chondrex; mouse IL-6, mouse TNF-α and mouse IL-1β ELISA kits from ABclonal; phosphorylated (p)-STAT6 antibody and p-JAK1 antibody were purchased from LifeSpan Biological Sciences and Cell Signaling Technology respectively; JAK1 antibody, anti-STAT6, anti-STAT1, p-STAT1 antibody, anti-GAPDH and anti-Vimentin antibodies were got from Abcam; TNF-α, IL-1β and IL-6 antibody were got from Abclonal; RiboFECT™ CP Transfection Kit were from RIOBIO; All real-time quantitative Polymerase Chain Reaction (PCR) reagents were purchased from OMEGA.

### Mice

Male DBA/1 mice (6–8 weeks) were purchased from Shanghai SLAC Experimental Animal Co. Ltd with production license No: SCXK 2017-0005. All the experiments were according to the ethical guidelines of the Research Ethics Committee of Shenzhen Peking University-The Hong Kong University of Science and Technology Medical Center. All animals were maintained under specific pathogen-free environment in the Center for Laboratory Animal Care, Shenzhen Peking University-The Hong Kong University of Science and Technology Medical Center with the guidelines and regulations for the use and care of animals.

### Induction and evaluation of CIA and SKN treatment

Male DBA/1 mice (6–8 weeks) were used and randomly divided into 4 groups with 6 mice in each group: control group, CIA model group (CIA group), the SKN treated group and the MTX treated group. Besides the control mice, all other mice were intradermally injected at the base of the tail with 100 μg bovine type II collagen (dissolved in 0.05 M acetic acid) emulsified 1:1 in CFA [18] on day 1, and boosted with 100 μg type II collagen in IFA on day 21.

After booster immunization, all mice were measured once every 1–2 days. And arthritis severity was measured by arthritis scoring with two independent, blinded experimenters according to a visual measurement swelling or inflammation and scored from 0 to 4 as published [19]. To quantify the severity of the disease, a macro scale from 0 to 4 was applied to each paw, 0, normal; 1, detectable arthritis with erythema; 2, obvious swelling and redness; 3, severe swelling and redness from joints to fingers; and 4, maximum swelling and deformity with ankylosis. The arthritis score per mouse was a cumulative value of all 4 limbs,and the maximum arthritis score could reach 16. Furthermore, the arthritis incidence rates are the arthritis mice number /total mice number in each group. SKN dissolved at 0.05% in DMSO and given intragastrically daily for a period of 20 days from the day after second immunization with syringe feeding. The selection for SKN (4 mg/kg/day) dosage was based on the data of a previous research [17]. MTX was intraperitoneally administered at a dose of 0.5 mg/kg and three times a week for 20 days. The selection for MTX (0.5 mg/kg) dosage is based on our previous pre-experiment. Meanwhile, equal volume of vehicle were given to CIA and control mice.

### Histological evaluation

For histological analyses, the mice were killed 40 days after the primary first immunization. The hind knees, liver, kidney and testis were achieved and dissected into sections for pathological measurement, and stained with hematoxylin and eosin (HE) while the front knees were used for western blot. As described previously, histopathologic severity was measured using three different parameters [20–22]: synovial hyperplasia, synovitis, cartilage damage and bone erosion. Each parameter was measured by two independent observers at 0–3 (0, normal; 3, severe).

### Histochemical analysis

After fixing, decalcification, slicing and dewaxing, put the slices in a glass jar, pour citric acid buffer, and ensure that the slices are always in citric acid buffer without drying; Antigen repair: repair by microwave oven; Blocking endogenous peroxidase: add an appropriate amount of endogenous peroxidase blocker (contained in the kit), incubate at room temperature for 10 min, and throw away the excess liquid; Incubation of primary antibody: according to the size of the tissue, add appropriate diluted primary antibody (TNF-α, IL-1β and IL-6 is diluted with primary antibody diluent 1:100 according to the instructions and exploration), and incubate in a wet box overnight; Dropping reaction enhancer: drop an appropriate amount of reaction enhancer, incubate at room temperature for 10 min, and throw away the excess liquid; Drip the Goat anti rabbit IgG polymer with enhanced enzyme label: drip an appropriate amount of Goat anti rabbit IgG polymer with enhanced enzyme label, incubate at room temperature for 10 min, and throw away the excess liquid; DAB color development. observe under the microscope, control the reaction time, 1–10 min, and wash with distilled water to terminate the reaction; Hematoxylin 20 s, washed twice with tap water; Hydrochloric acid and alcohol for 2 s, washed with tap water for 2 times; Ammonia back blue for 16 s; Seal the dehydrator. After taking photos, the integrated optical density (IOD) was analyzed by image pro plus.

### Cell culture

FLS were separated from synovium of RA patients with synovectomy or joint replacement surgery in Peking University Shenzhen Hospital (Shenzhen, China), who fulfilled the 1987 American College of Rheumatology criteria for RA [23], from March 2017 to September 2018. All patients were informed about the aims of specimen collection and given the signed written consent. This research met the standards of the local human ethics committee, and getting informed consent of all participants. Primary cultures set up after the fresh synovium being isolated enzymatically, according to the method mentioned before [24] with minor modification. Cold phosphate-buffered saline (PBS) rinsed the synovial tissue if contaminated with blood, and moved to a 100-mm Petri dish. The synovium was removed and rinsed 3 times with PBS. Tissue was then placed in a new 100-mm Petri dish and inspected—it contained fibrous membranes, mixture of synovium, fat, and possibly cartilage and/or bone fragments. Synovium, present on the inner aspect of the joint capsule, appeared pink or tan, sometimes villous, and was easily distinguishable from yellow/white fat and fibrous tissue. Synovial tissue was identified, excised with surgical scissors, and placed in a fresh Petri dish. Excised synovium was minced with sterile scissors until individual fragments were 1–2 mm^3^. Minced tissue was placed in the bottom of a 250 mL flask, then gently turned upside down on the bottom of the flask and about 4 mL of culture fluid was introduced into the flask, before culture in an incubator for 2–4 h to adhere the tissue. The culture flask was reversed and laid flat and stationary. A little DMEM medium which including 20% fetal bovine serum was mixed to cover the tissue mass before continued culture in the incubator. The nutrient solution was changed every 1 to 3 days. After FLS had grown into the tissue blocks, they were removed and continued to be cultured. Then FLS were seeded in sterile DMEM companied with 100 U/mL penicillin, 10% FBS, 80 U/mL streptomycin (called complete medium later), and cultured in a humidified 5% CO_2_ incubator at 37℃. In this study, FLS were used at passage numbers 4 to 8.

### Immunofluorescence

To measure FLS in the inflamed joint synovium in vivo and FLS cells in vitro, polyclonal antibody recognizing the vimentin/SOCS1 pan-endothelial antigen was used on knee joint tissue cut into 5 μm thick paraffin-embedded sections for immunofluorescence analysis. The sections were incubated overnight in 4℃ for vimentin (dilution of 1:10) and SOCS1 (dilution of 1:50), and then incubated with secondary antibody (dilution of 1:400) for 1 h at room temperature. At 400 × magnification, the average ROI represents the percentage of area covered by positive staining cells in each image. After dual staining for vimentin and SOCS1, the cells were assessed using Image Pro-Plus.

### Cell viability assay

In 96-well plates, FLS (5 × 10^4^ cells/mL) were inoculated into each well and cultured in sterile complete H-DMEM medium for 24 h, 10% FBS, and 80 U/mL streptomycin. Where after, cells were cultured in the existence of SKN (0.05, 0.1 and 0.2 μM) for 24 h. Viability of cells was measured by the MTT method. There were three measurements in our experiment.

### Scratch healing assay

The experiment was carried out as described previously [21, 22, 25]. FLS (5 × 10^4^ cells per well) were cultured in a 48-well plate and incubated overnight yielding confluent monolayers for wounding. A pipette tip was used to make wounds and immediately taking photographs for the wounds(time zero). Then 100 μL of sterile complete H-DMEM medium, with or without 20 ng/mL TNF-α, and different concentrations of SKN (0, 0.05, 0.1 and 0.2 μM), were added to the wells after washing with PBS. After 12 h later, these wounds were taken photographs again. The experiments were done three times independently.

### Transwell migration assay

The migration of FLS to TNF-α as mentioned earlier, Transwell migration analysis was used [21, 22, 25]. In the lower wells, 500 μL complete H-DMEM contained with 20 ng/mL TNF-α prepared was placed. And 1 × 10^4^ FLS cells suspended in sterile H-DMEM medium containing SKN (0.05, 0.1 and 0.2 μM) were inoculated in the upper wells and cultured at 37 °C for 6 h. Carefully remove the not migrating cells from the upper pore with a cotton swab, and FLS on the lower surface of the membrane were fixed and stained with 0.1% crystal violet solution. Then the total counts of migrated cells in each insert were measured in five randomly selected fields by optical microscopy (400 × magnification). All experiments were carried out independently in triplicate.

### Cell invasion assay

This experiment was carried out in Transwell room, of which the transwell insert upper surfaces were pre-coated with 1.25 mg/mL Matrigel (20 μL/well) at 37 °C for 30–60 min and the bottom chamber contained 600 μL of culture medium with or without 20 ng/mL TNF-α as mentioned previously [21, 22, 25]. 1 × 10^4^ cells/well FLS were seeded on the upper chamber and incubated in normal H-DMEM medium with different concentrations of SKN (0, 0.05, 0.1 and 0.2 μM). After 14 h of culture, the invasive cells were fixed and stained with 0.1% crystal violet solution, while non-invasive cells on the upper membrane surfaces were wiped by cotton swabs. Cell invasion was evaluated by counting the number of cells on the lower surface with a phase-contrast microscope. And the migrating cell numbers were counted in six random fields. All the tests were carried out three times.

### Cell adhesion assay

FLS (5 × 10^4^ cells/mL) were cultured in the pore of 24-well plates as previously described [21, 22, 25], and then cultured with or without 20 ng/mL TNF-α, and different concentrations of SKN (0, 0.05, 0.1 and 0.2 μM) for 24 h. Treated/untreated cells were then seeded in 20 mg/L fibronectin (FN), or 10 mg/mL bovine serum albumin (BSA, used as negative control)-coated 96-well plates and incubated in sterile complete H-DMEM medium for 1.5 h. Then cells were washed twice with PBS, followed by sterile H-DMEM (200 μL) including 5% FBS and 10% (v/v) MTT reagent adding to the cells and measuring with a microplate reader at 490 nm. The results were expressed as cell adhesiveness. All tests are in triplicate.

### Enzyme-linked immunosorbant assay (ELISA)

We collected the sera from control, CIA, and SKN-treated CIA mice. The expression of TNF-α, IL-6, and IL-1β in sera were measured by ELISA according to specifications. All assays were carried out in triplicate.

### Real-time PCR

FLS (5 × 10^4^ cells/ml) were seeded in 6-well plates (2 ml cells/well) and incubated in sterile H-DMEM supplemented with 10% FBS, 100U/ml penicillin and 80 U/ml streptomycin for 24 h. Si-SOCS1 was added for 48 h, and then FLS were incubated and plus different concentrations of SKN (0.05, 0.1 and 0.2 μM) for 24 h. Total cell RNA was extracted using RNA purification kits purchased from Invitrogen in accordance with the manufacturer’s instructions. Real time-PCR reactions were run in a Light Cycler 96 system. Reaction cycle conditions were as follows: 42℃ for 2 min, 37℃ for 15 min and 85℃ for 5 s of pre-denaturation conditions, 40 cycles at 95℃ for 15 s and 60℃ for 20 s and 72℃ for 30 s. The primer sequences of the genes were showed as Table 1. The ratios of the mRNAs examined against GAPDH were obtained and expressed as mean ± SEM.

**Table 1:** Primer Sequence

Gene	Species	Types	Sequences
SOCS1	Human	Forward	CACTTCCGCACATTCCGTTC
		Reverse	AGGCCATCTTCACGCTAAGG
GAPDH	Human	Forward	CACTAGGCGCTCACTGTTCT
		Reverse	GCGCCCAATACGACCAAATC

### Western-blot analysis

The assay was measured as previously mentioned [21, 22, 25]. Synovium tissue of mice knee joints were collected. FLS were pre-treated for 1 h with 20 ng/mL TNF-α, and cultured with different concentrations of SKN (0, 0.05, 0.1 and 0.2 μM) for 24 h before cell preparation. Meanwhile, FLS were pre-treated with SKN for 2 h and subsequently stimulated for 15 min with 20 ng/mL TNF-α before cell collection. Lysis buffer was used to extract total protein. Cell lysates (50 μg) were loaded and separated by 10% sodium dodecyl sulfate–polyacrylamide gel electrophoresis, subsequently blotted onto a polyvinylidene fluoride membrane, and then blocked for 2 h at room temperature and incubated overnight at 4 °C with primary antibodies for p-JAK1 (dilution 1:500), JAK1 (dilution 1:1000), p-STAT1 (dilution 1:500), STAT1 (dilution 1:500), p-STAT6 (dilution 1:200), STAT6 (dilution 1:200), and GAPDH (dilution 1:2500). Then, membranes were incubated with secondary antibodies conjugated to horseradish peroxidase for 1 h at room temperature. The GAPDH expression level was regarded as an internal standard. The experiments were carried out three times.

### Molecular docking

Docking studies were put into effect by using Autodock Vina program [26]. Docking was carred ou to obtain a population of possible conformations and orientations for the ligand at the binding site. The protein was converted to PDBQT file that contains a protein structure with hydrogens in all polar residues. All bonds of ligands were set as rotatable. All calculations for protein-fixed ligand-flexible docking were done using the Lamarckian Genetic algorithm (LGA) method. As for the proteins, the point numbers equal to 100 in the directions X and Y, and 110 in the direction Z were used to prepare the grid box to cover the whole protein structure. After the docking search completed, the best conformation was chosen with the lowest binding energy. The interactions complex protein–ligand conformations, containing hydrogen bonds and the bond lengths were analyzed using PyMol.

### Statistical analysis

Statistical analysis used SPSS version 11.0 software for Windows. Continuous variables were presented as means ± SEM. Non-parametric Kruskal–Wallis tests were used to analyze pathological scores, and ANOVA followed by a post hoc test or Student’s t-test for other data. *P* < 0.05 was statistically significant.

## Results

### SKN alleviates arthritis progression and disease severity of CIA mice

**Fig. 1:**
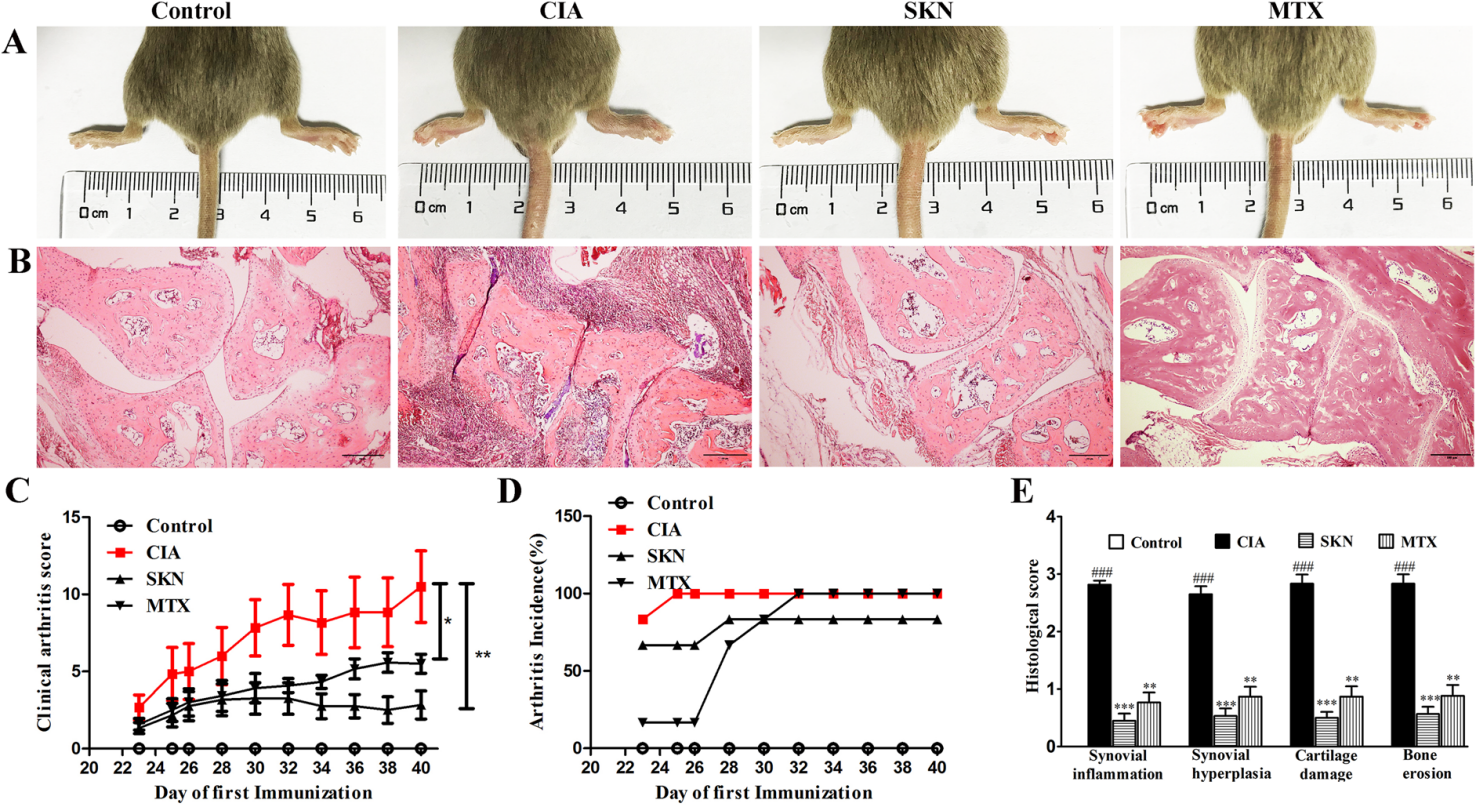
Therapeutic roles of SKN on collagen-induced arthritis (CIA) mice. CIA mice were intragastric administered with 4 mg/kg SKN, or vehicle for 20 days from the day of booster immunization. **A** Macroscopic measurement of arthritis like erythema or swelling was assessed in mice paws of all groups. **B** HE staining images of metatarsophalangeal joint sections of each group. **C** Arthritis score was evaluated with a scale of 0–4 for each paw, and a possible maximum score of 16 for each mouse. **D** Arthritis incidence rate are the positive number/total number in every group. **E** The evaluated and analyzed histological scores of each group. The data is expressed as an average ± SEM (n = 6). *P < 0.05, **P < 0.01, ***P < 0.001, compared with the CIA group

The CIA model of DBA/1 mice was used to observe the effect of SKN on arthritis. Daily treatment of SKN was started from day 21 to day 40 in which the first immunization was made. Macroscopic evidence of arthritis, like swelling or erythema, was marked increased in the CIA group, and SKN markedly reduced joint inflammation in CIA mice (Fig. 1A). The same of the score of arthritis, arthritis incidence, clinical symptoms, and the histopathological evaluation also showed that SKN was observably effective (Fig. 1B and E). As shown that in Fig. 1C and D, SKN alleviated the increasing score of arthritis and arthritis incidence from day 23 after first immunization in CIA mice. We also found that cartilage damage, inflammation and bone erosion were markedly reduced in SKN treatment group, and SKN exerted a better effect than MTX as showed in the histopathological evaluation. Moreover, we demonstrated that SKN has no obvious hepatotoxicity and reproductive toxicity measured by HE (Additional file 1). All in all, these datas show that systemic administration of SKN in mice reduces the clinical and pathologic severity of CIA. Moreover, the effect of SKN is better than that of the positive control, MTX.

### SKN inhibits synovitis in synovial joint tissue in CIA

**Fig. 2:**
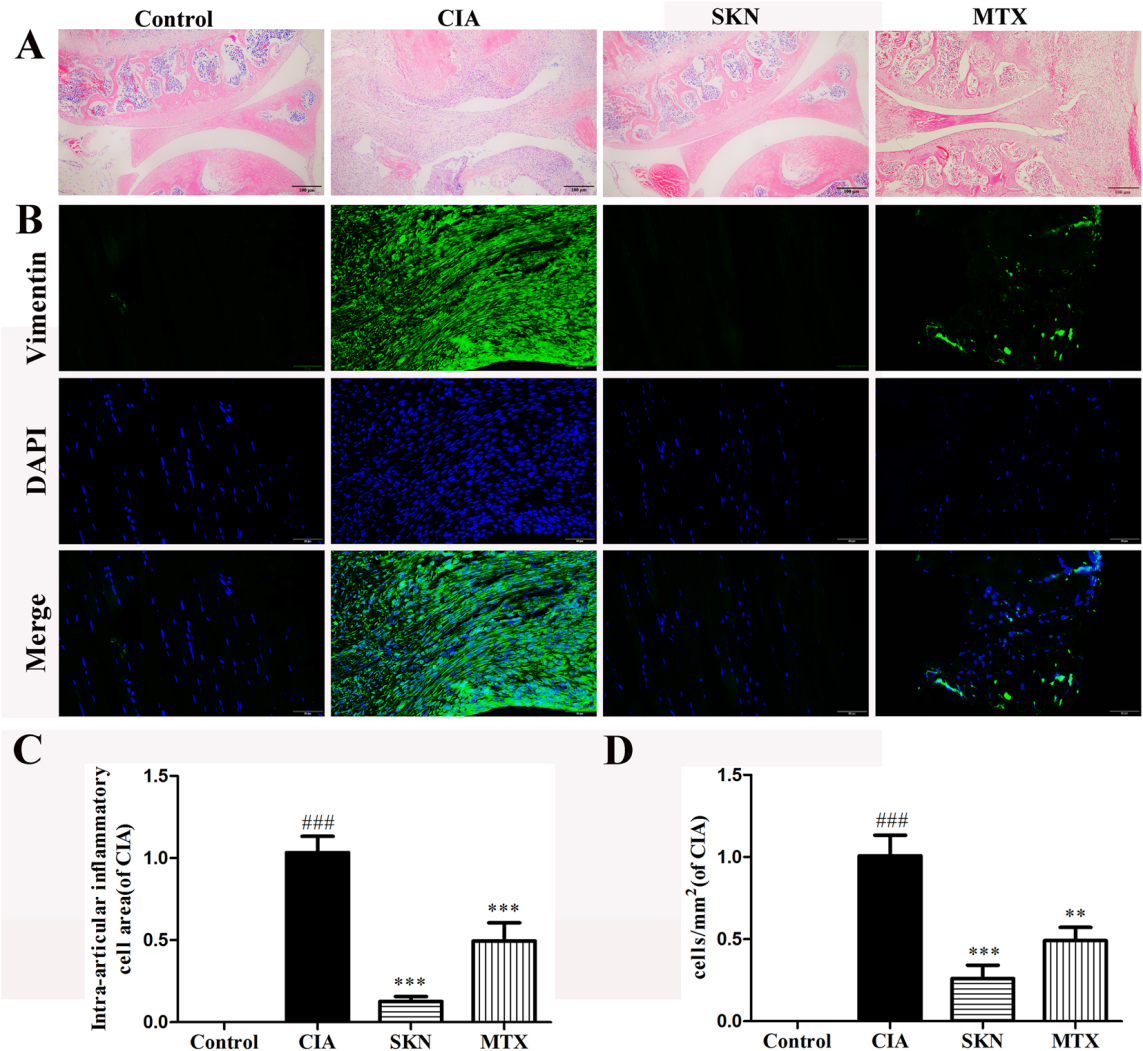
SKN reduces the inflammatory cells density in the synovium of inflamed joints in collagen-induced arthritis (CIA) mice. **A**, **C** HE staining photomicrographs of synovium from knee joints of normal control, CIA, SKN (4 mg/kg)-treated CIA mice and MTX (0.5 mg/kg)-treated CIA mice, respectively. **B**, **D** Vimentin immunofluorescence staining images of synovium from knee joints of normal control, CIA, SKN (4 mg/kg)-treated and MTX (0.5 mg/kg)-treated CIA mice are shown, respectively. Data is expressed as an average ± SEM (n = 6). ^###^p < 0.001, compared to the control group; *P < 0.05, **P < 0.01, and ***P < 0.001, relative to the CIA group

In the development of RA, synovitis is a important step in progression towards chronic arthritis. As shown in Fig. 2A and C, the extent of synovitis was inhibited by a dose of 4 mg/kg SKN, as the mean synovial inflammatory cell density reduced compared to the CIA group. Meanwhile, immunofluorescence was used to detect expression of vimentin, a marker of FLS in the synovium. Our results showed that a significant number of vimentin staining was present in synovium of inflamed joints from CIA mice, and the same situation was obviously attenuated in SKN-treated and MTX-treated mice (Fig. 2 B and D).

### SKN decreases expression levels of pro-inflammatory mediators

**Fig. 3:**
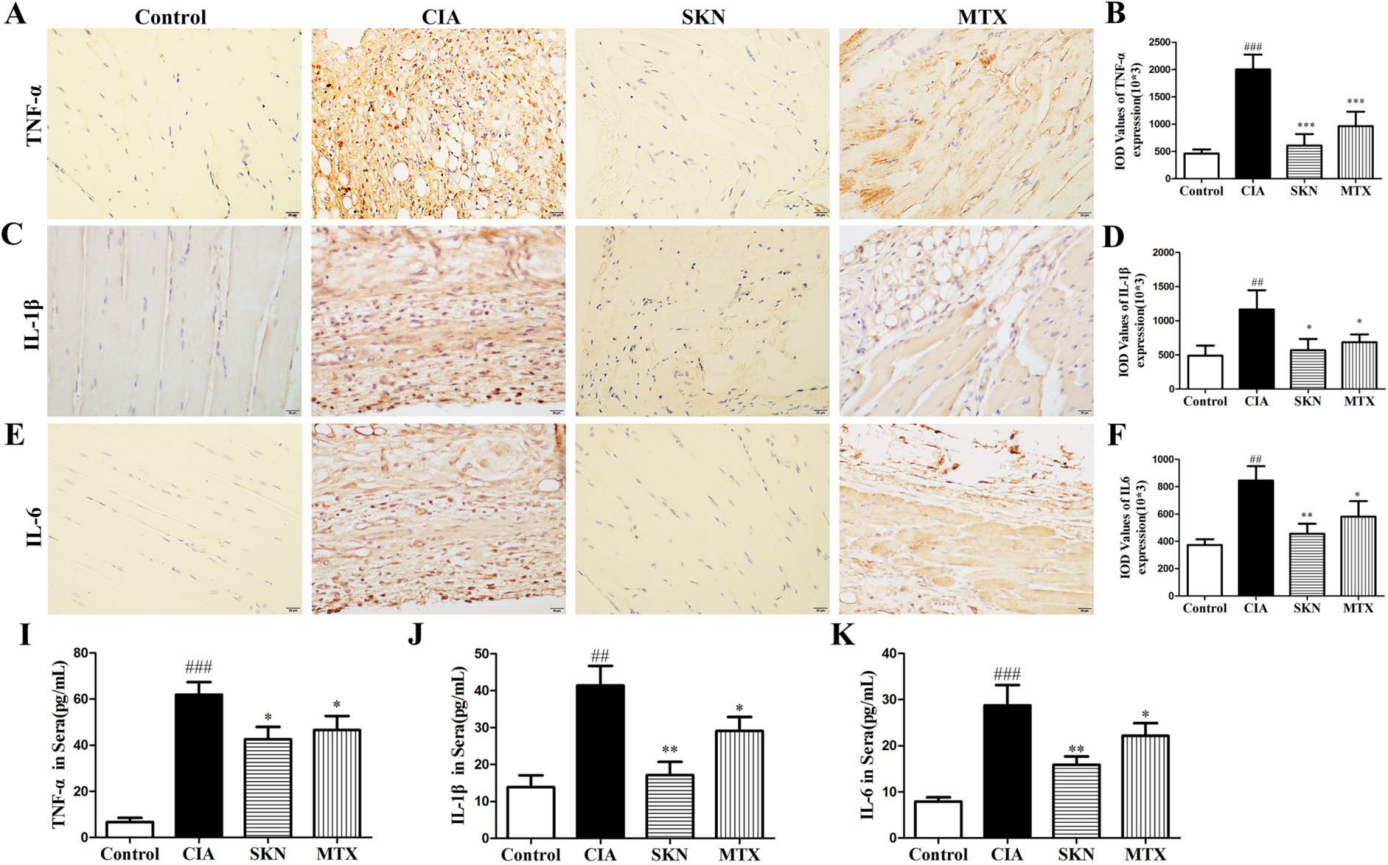
SKN inhibits expression levels of pro-inflammatory mediators in CIA mice. From the day of intensive immunization, CIA mice were given 4 mg/kg SKN and 0.5 mg/kg MTX for 20 consecutive days. The expression of TNF-α (A,B,G), IL-1β(C,D,H), IL-6 (E,F,I) in synovium and serum of knee joints of mice. The experiments were carried out in triplicate indenpendently and calculated Mean ± SEM (n = 6). ***P < 0.001, **P < 0.01, and *P < 0.05, compared with the CIA group; ^##^*P* < 0.01, ^###^*P* < 0.001, compared with the control group

Next, we measured the protein levels of pro-inflammatory mediators including TNF- α, IL-1β and IL-6 were detected in joint by IHC and in mice sera by ELISA. Our result showed that SKN strongly reduced TNF-α, IL-6 and IL-1β (Fig. 3) expression in synovia and sera of CIA mice, suggesting that SKN could decrease RA synovitis, and SKN exerted a better effect than MTX.

### Cell Morphology

**Fig.4:**
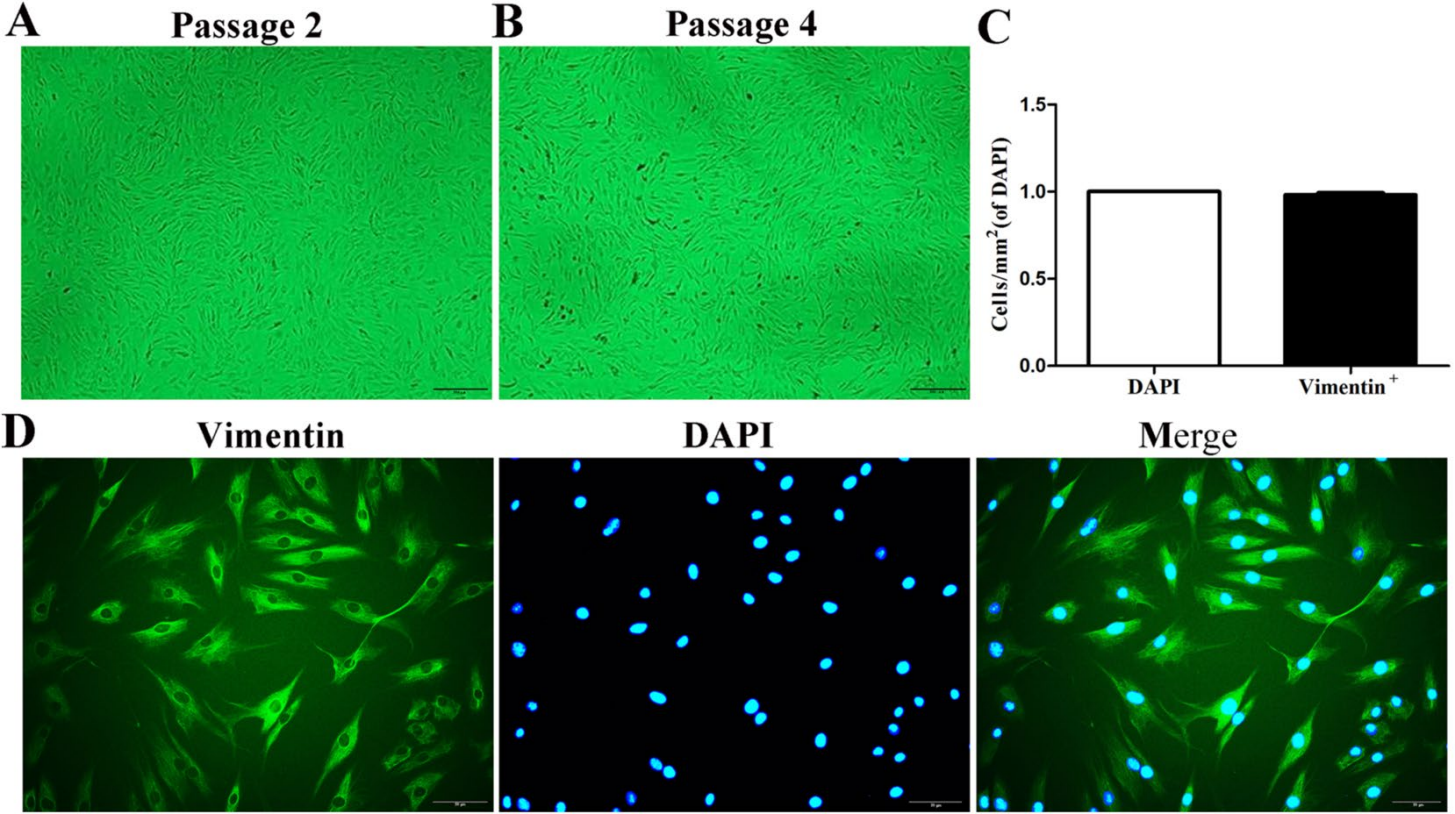
Cell Morphology of FLS. **A** Cell Morphology of the second generation FLS. **B** Cell Morphology of the forth generation FLS. **C**, **D** The positive rate of Vimentin in FLS

In order to explore the underlying mechanism of SKN in the treatment of RA synovitis, we primary cultured FLS appeared synovial tissue of RA patient, with cells growing in a radial distribution (Additional file 2 A). With the passage of time, this out-growth became more obvious (Additional file 2 B, C). After passage (the second generation), FLS became larger with more extensions (Fig. 4 A). The fourth generation of FLS were essentially fibroblast-like cells (Fig. 4B)—cell morphology was regular and spindle-shaped. Nuclei were oval and in the center of the cell, with cells appearing healthy. Immunofluorescence results showed that the percentage of vimentin positive cells in synovium was over 98% (Fig. 4C, D), indicating that the cells we cultured were FLS.

### SKN inhibits the migration, invasion and adhesion of FLS

**Fig. 5:**
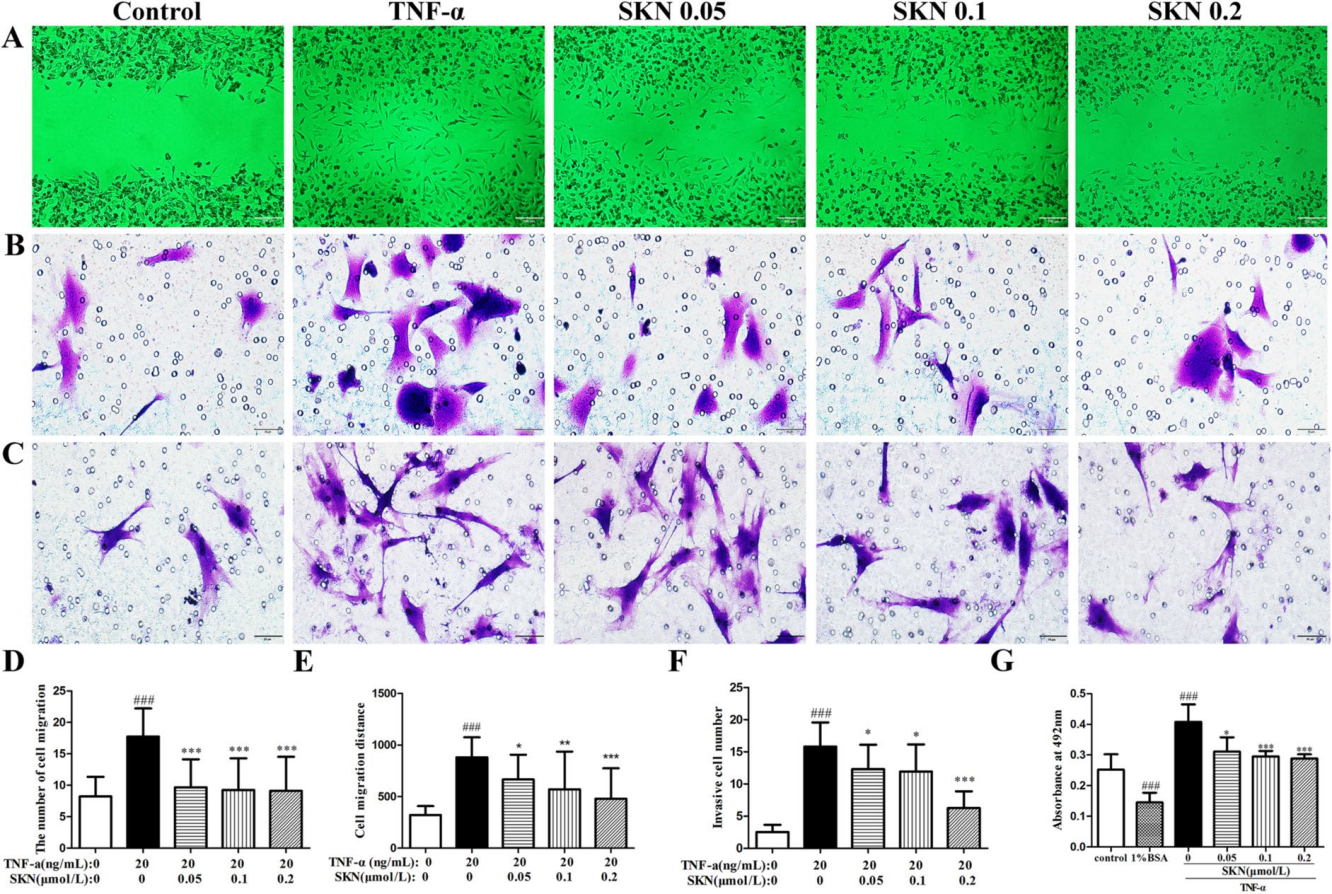
SKN suppresses TNF-α-induced FLS migration, invasion, and adhesion. **A**, **D** FLS which seeded in 48-well plates were scratched with pipette tips for wound, then allowed to migrate in the presence of 20 ng/ml TNF-α with or without SKN (0.05, 0.1 and 0.2 μM) for 12 h. The distance of cell migration was calculated. **B**, **E** FLS were presented in upper insert with serum-free H-DMEM and TNF-α were put in lower insert with 10%FBS H-DMEM. The migration cell number were counted after 6 h in the presence of SKN (0.05, 0.1 and 0.2 μM) or not. **C**, **F** FLS were presented in Matrigel matrix pre-coated upper chamber with serum-free H-DMEM, migrated for 14 h in the presence of 20 ng/ml TNF-α with SKN (0, 0.05, 0.1 and 0.2 μM), then calculate the number of intruders. **G** FLS were seeded on the 24-well plates with or without the presence of 20 ng/ml TNF-α and/not SKN for 24 h, and then plated the treated cells on fibronectin or bovine serum albumin for 1.5 h, the cell viability by MTT. The experiments were carried out in triplicate indenpendently and calculated Mean ± SEM (n = 3). *P < 0.05, ** *P* < 0.01, *** *P* < 0.001, compared with the TNF-α-induced group; ^###^*P* < 0.001, compared to the control group

FLS migrate in response to many chemotactic factors in synovitis. So that, we tested whether SKN regulates TNF-α-induced the migration, invasion and adhesion of FLS. As shown that in Fig. 5 A and D, SKN inhibited FLS wounding migration that induced by TNF-α in a dose-dependent manner. Furthermore, SKN markedly inhibited the TNF-α-directed FLS chemotaxis in a dose-dependent manner, determined with a transwell chamber (Fig. 5B, E). Transwell chamber which was pre-coated with Matrigel experiments were also done to measure the role of SKN on endothelial cell invasion, and SKN dose-dependently lowered invasive cells number migrating to the underside of the filters after TNF-α stimulation (Fig. 5C, F), indicating a potential inhibitory role of SKN on TNF-α-induced FLS invasiveness. In addition, the role of SKN on cell adhesiveness of FLS was tested by adhesion assay. SKN markedly inhibited the cell adhesiveness of FLS induced by TNF-α at a concentration ranging from 0.05 to 0.2 μM (Fig. 5G). Furthermore, to confirmed whether the above suppressive role of SKN was due to cytotoxicity, FLS was cultured with SKN and/or TNF-α for 24 h, then measured by MTT assay for cell cytotoxicity detection and by flow cytometry for cell cycle and apoptosis. Our results indicated that SKN shown non-cytotoxic effects on FLS in our present study (Additional file 3, Additional file 4, Additional file 5), advising that SKN specifically inhibits the above functions of FLS. We also calculated the IC_50_ of SKN on FLS is 32.48 μM.

### SKN might interact with SOCS1

**Fig.6:**
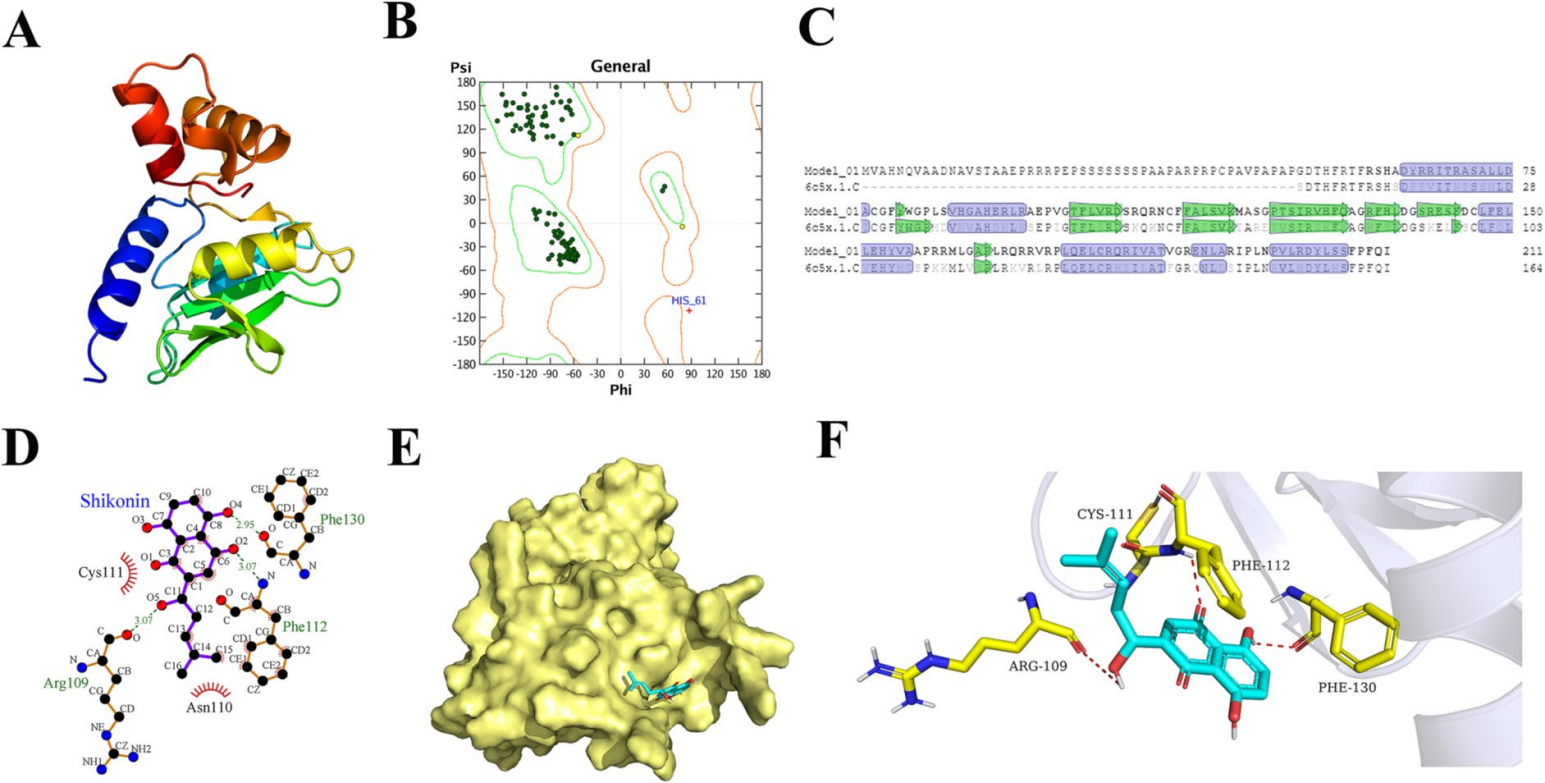
SKN might interact with SOCS1. **A** The homology model of SOCS1. **B** Ramachandran plot for SOCS1. Dark green dots represent the residues in favored regions; yellow dots represent the residues in allowed regions, red cross represent the residues in irrational regions. **C** The structure-based sequence comparison between SOCS1 and the template structure. The mismatched residues are displayed with fade, the sequence corresponding to alpha-helix and beta strand regions are marked with blue and green color. **D** The 2D binding mode of SOCS1 and SKN. **E** The binding model of SKN on molecular surface of SOCS1. SKN is colored in cyan, the molecular surface of SOCS1 is colored in pale yellow. **F** The 3D binding mode of SOCS1 and SKN. SKN is colored in cyan, the surrounding residues in the binding pockets are colored in yellow, the backbone of the receptor is depicted as white cartoon with transparency

In order to get the potential mechanism of SKN in inhibiting FLS, we screened the target protein of SKN using molecular docking. As shown in Table 2. the docking score of SKN and SOCS1 is -6.0 kcal/mol, suggesting that SKN might interact with SOCS1. The modeling result of SOCS1 was depicted in Fig. 6 A and B. The Ramachandran plot for SOCS1 showed that 99% residues are in allowed regions, indicating that the 3D structure of the model is reasonable. The structural analysis of the SOCS1 modeling results were shown in Fig. 6C. The SOCS1 structure is basically consistent with the template structure. Both of them have the same alpha helix and beta strand regions. The overall identity of the amino acid sequence was 63.98%. The binding model between SOCS1 and SKN were shown in Fig. 6D–F. SKN has formed a suitable steric complementarity with the binding site of SOCS1. Otherwise, hydrogen bond interactions and ion contact were formed among SOCS1 and SKN. The oxygen atom (O2) of SKN, regarded as hydrogen bond acceptor, forms hydrogen bond with the nitrogen atom (N) of Phe112. The oxygen atom (O4) of SKN, regarded as hydrogen bond donor, forms hydrogen bond with the oxygen atom (O) of Phe130. The oxygen atom (O5) of SKN, regarded as hydrogen bond donor, forms hydrogen bond with the oxygen atom (O) of Arg109. VDW interactions were also formed among SKN and the surround residues. These interactions mainly contribute to the binding energy between SOCS1 with SKN.

**Table 2:** The docking score of shikonin with SOCS1

Receptor	Ligand	Docking score (Kcal/mol)
SOCS1	Shikonin (SKN)	− 6.0

### SKN targeted on SOCS1 in TNF-α-induced FLS and CIA mice

**Fig. 7:**
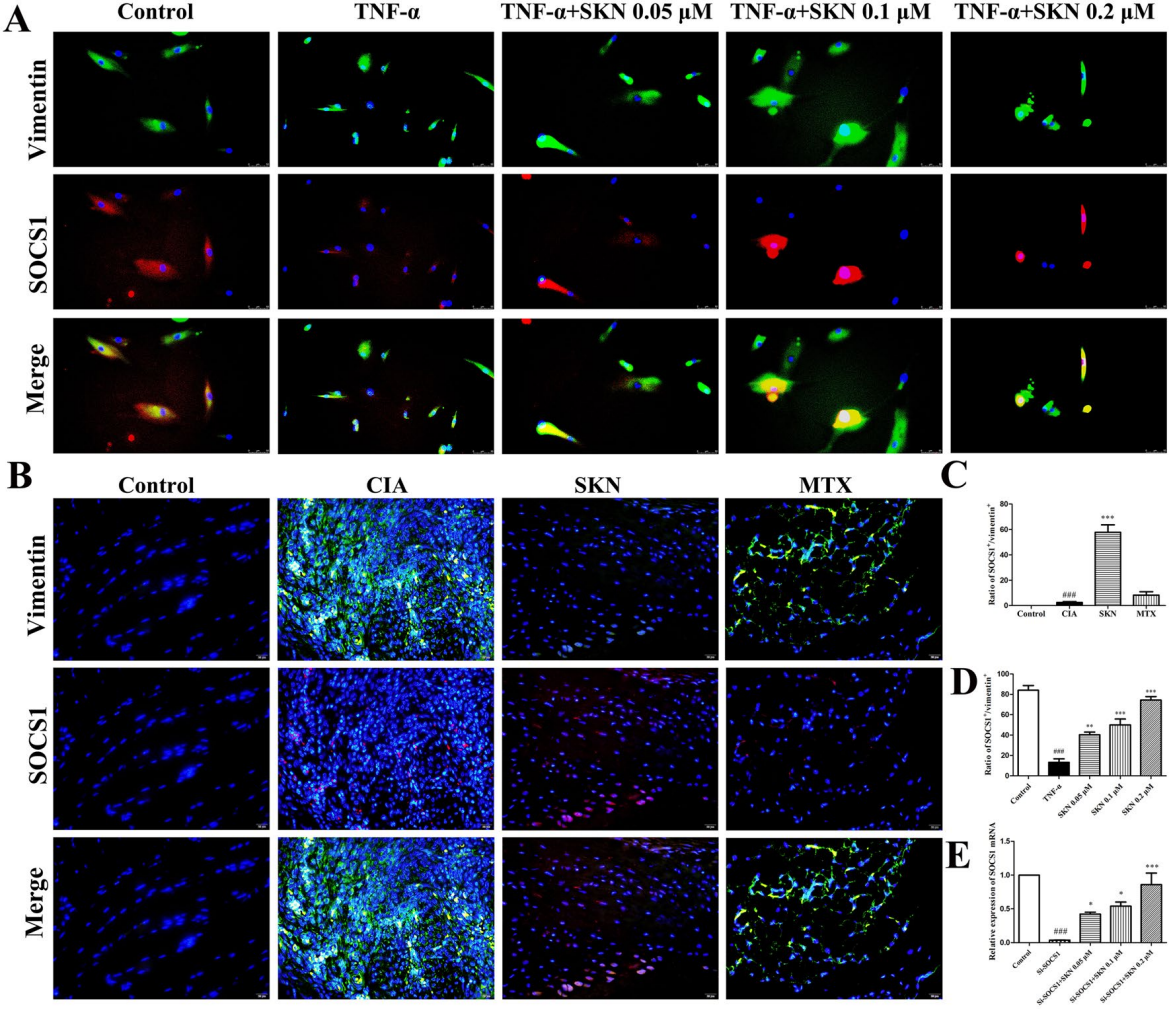
SKN targeted on SOCS1 in TNF-α-induced FLS and CIA mice. **A**, **D** FLS which seeded in 48-well plates in the presence of 20 ng/ml TNF-α with or without SKN (0.05, 0.1 and 0.2 μM) for 24 h and tested by IF. The number of vimentin + SOCS1 + cells were counted. **B**, **C** The number of vimentin + SOCS1 + cells was less in the joints of CIA mice of CIA group, while it was more in SKN group. At the same time, we silenced the SOCS1 gene in FLS. PCR results showed that shikonin could significantly increase the expression of SOCS1 in FLS silencing SOCS1 (Fig. 7E). All these indicate that shikonin acts on SOC in synovial cells. The experiments were carried out in triplicate indenpendently and calculated Mean ± SEM (n = 3 in vitro or 6 in vivo). *P < 0.05, ** *P* < 0.01, *** *P* < 0.001, compared with the TNF-α-induced /CIA group group; ^###^*P* < 0.001, compared to the control group. ***P < 0.001, **P < 0.01, and *P < 0.05, compared with the TNF-α-induced /CIA group; ^##^*P* < 0.01, ^###^*P* < 0.001, compared with the control group

In order to verify that the target of shikonin is SOCS1, we first used immunofluorescence in FLS and in CIA mice. The results show that vimentin^+^SOCS1^+^ cells was decreased in the FLS of TNF-α group (Fig. 7 A, D)and in the joints of CIA mice of CIA group, while it was markedly increased in SKN group(Fig. 7B, C). At the same time, we silenced the SOCS1 gene in FLS. PCR results showed that SKN could significantly increase the expression of SOCS1 in FLS silencing SOCS1(Fig. 7E). All these indicate that shikonin acts on SOCS1 in synovial cells.

### SKN restrains the activation of JAK/STAT pathway in TNF-α-induced FLS and CIA mice

**Fig. 8:**
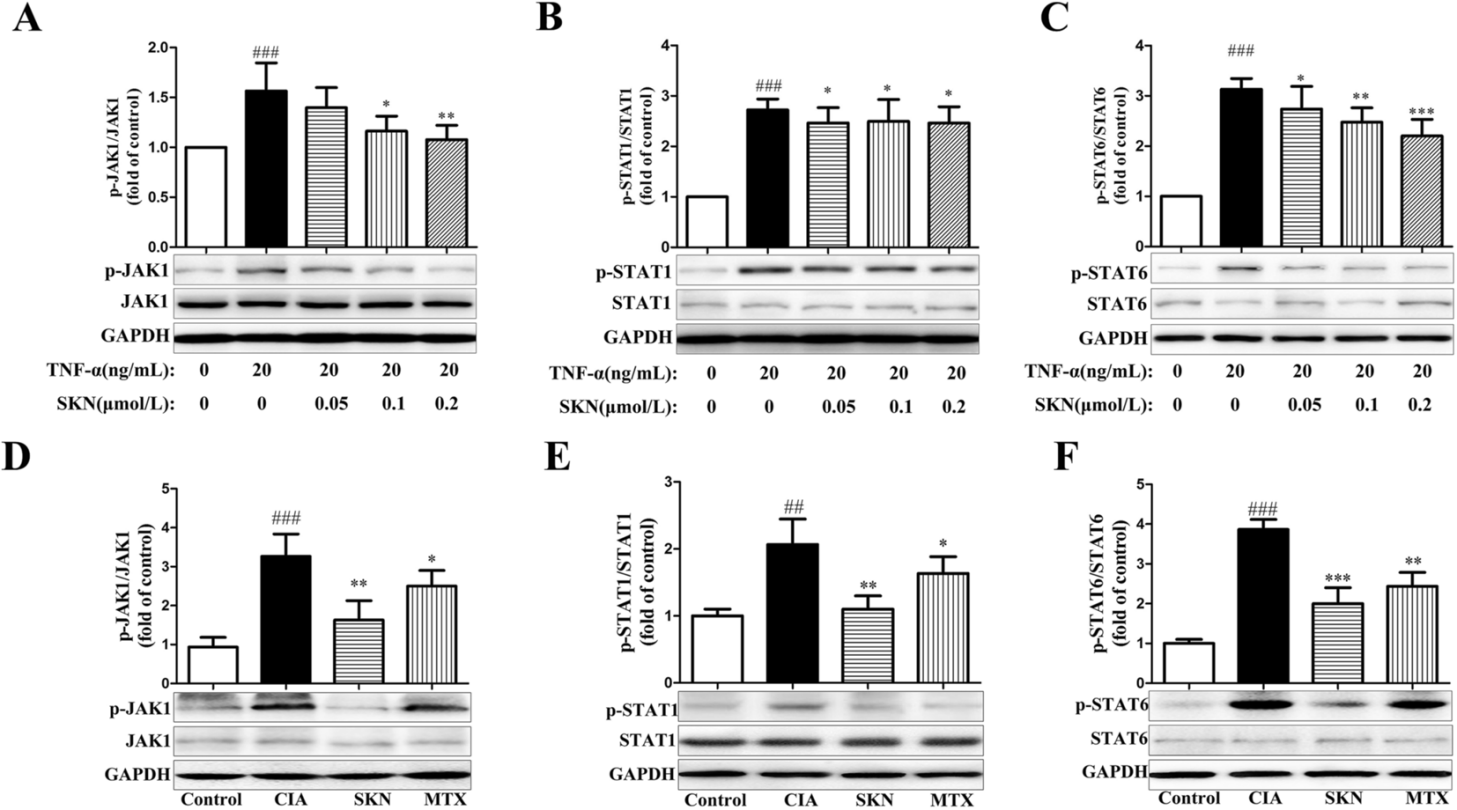
SKN inhibits the activation of JAK/STAT pathway in TNF-α-induced FLS and CIA mice. FLS for p-JAK1, p-STAT1 and p-STAT6 were starved for 48 h in 10% FBS medium, pretreated with SKN for 2 h,and then stimulated with 20 ng/ml TNF-α for 15 min before collection. The phosphorylations of JAK1 (**A**), STAT1 (**B**), and STAT6 (**C**) in FLS were measured by western blot. From the day of intensive immunization, CIA mice were given 4 mg/kg SKN and 0.5 mg/kg MTX for 20 consecutive days. The levels of p-JAK1 (**D**), p-STAT1 (**E**), and p-STAT6 (**F**) in joint of CIA mice treated with SKN were analyzed by western blot, too. The experiments were carried out in triplicate indenpendently and calculated Mean ± SEM (n = 3 for in vitro assays, n = 6 for in vivo assays). ***P < 0.001, **P < 0.01, and *P < 0.05, compared with the CIA/TNF-α-induced group; ^##^*P* < 0.01, ^###^*P* < 0.001, compared with the control group

As SOCS1 regulates JAK/STAT pathway, we futher confirmed the effect of SKN on the activation of JAK1, STAT1 and STAT6. Our research showed that SKN significantly inhibited the expression of p-JAK1, p-STAT1, p-STAT6 (Fig. 8A–C) in TNF-α-induced FLS. In addition, the levels of p-JAK1, p-STAT1 and p-STAT6 (Fig. 8D–F) in joint of SKN-treated CIA mice were also obviously decreased. These findings indicated that SKN inhibits the activation of JAK/STAT pathway in TNF-α-induced FLS and CIA mice. Moreover, the effect of SKN is stronger than MTX.

## Discussion

RA is a autoimmune, common and inflammatory disease which is with unknown cause, that is associated with progressive disability, systemic complications, and mortality—prognosis is guarded [27]. Synovitis hyperplasia and angiogenesis are two important pathological links in RA, which play an important role in the progress of RA [28]. Our previous research have proved that angiogenesis plays an significant role in RA, SKN and Ermiao San can effectively inhibit synovial angiogenesis in RA [20, 21]. Some studies have confirmed that SKN can act on synovial cells [5, 6], but its specific target is unclear, which is the focus of our article.

FLS are the pathogenic cell of RA and the main effector cells of synovitis, which play a crucial role in the pathogenic development of RA. FLS were characterized by their resistance to apoptosis, resulting in over-expansion and their destruction of articular cartilage, which were also called synovial lining fibroblasts and [29]. FLS conduce to the local production of cytokines, the protein mediators of inflammation, and proteolytic enzymes that degrade the extracellular matrix in RA [30]. Therapies targeting FLS should improve the efficacy of clinical treatment of inflammatory arthritis without suppressing systemic immunity [31]. To inhibit synovial tissue hyperplasia is the main aim of RA treatment as it forms the pannus which could irreversibly destroys joint cartilage and bone [32]. Studies have shown that the biological characteristics of RA synovial cells are changed under stimulation from inflammatory factors, and proliferation, migration and invasion of RA synovial cells is greatly enhanced [33, 34]. In this study, we used TNF-α-induced FLS as an in vitro model. Our results showed that TNF-α could significantly enhance the proliferation, migration, adhesion and invasion of FLS at a concentration of 20 ng/mL, while SKN at doses of 0.05–0.2 μM could significantly reduce the adhesion, invasiveness and migration of FLS induced by TNF-α.

FLS present a main role in the pathogenesis of RA as effector cells, producing inflammatory cytokines, chemokines and matrix metalloproteinases [35]. The essential role of cytokines, specifically IL-1β and TNF-α, in the development of RA is well proved now [36]. Successful clinical applications of IL-1β and TNF-α blockade in RA has led to crucial interest in regulating production of IL-1β and TNF-α and targeting the transcription factors and signaling pathways utilized by IL-1β and TNF-α^37^. TNF-α is existed in RA synovium and directly promots FLS proliferation and activation, a pivotal event in the pathogenesis of RA [38]. IL-6 is considered a critical cytokine that drives inflammatory joint destruction in RA. IL-1β is an important pro-inflammatory cytokine. It contributes to the inflammatory proliferation of synoviocytes involved in the pathogenesis of RA [31]. In this study, we observed that TNF-α induced FLS to secrete high levels of IL-1β, and SKN at doses of 0.05–0.2 μM could significantly inhibit this effect. These results suggest that SKN can inhibit inflammation and proliferation of synovial cells by inhibiting IL-1β secreted by FLS induced by TNF-α. Moreover, SKN significantly reduced the protein level of IL-6, IL-1β and TNF-α in CIA mice. Taken together, our data indicate that SKN inhibits synovitis by downregulating pro-inflammatory factors, including IL-1β, TNF-α and IL-6.

The synovium is infiltrated by the synovial fluid and leukocytes is enriched with pro-inflammatory mediators that could induce an inflammatory cascade, which is featured with interactions between FLS and cells of the innate immune system. In RA, the FLS dysfunction leads to a hyperplastic synovium. The abnormal FLS proliferation results from a loss of contact inhibition that plays a inportant role in RA through inducing inflammatory mediators. Many cytokines use JAK and STAT pathway to exert their effects in the pathology of RA [39]. The JAK/STAT pathway plays an significant role in cytokine and growth factor-induced signal transduction. Signaling through this pathway is mediated by phosphorylation of STAT proteins [40]. SOCS1 is one of the most important members of the SOCS protein family which is a negative regulator of JAK/STAT signaling and mainly exerts its effects on inflammation, autoimmune diseases and other diseases through the JAK/STAT pathway [6]. In the current study, we found SOCS1 might be the target protein of SKN. A large number of studies have confirmed that the JAK/STAT pathway is involved in regulating the production and activation of cytokines in the synovium of the RA joint and in the pathophysiological process within the RA joint.

A wide range of cytokines, including amongst others IL1β, IL-6 have been found to stimulate the induction of hyperproliferation,and cytokines binding with its receptors on effector cells, by means of intracellular enzymatic signal transduction cascades such as the JAK/STAT pathway, can lead to cells activation, proliferation and so on [41]. Many cytokines, such as TNF-α and IL-1, are responsible for the inflammation of RA by activating JAK1 pathways. In addition, some studies have reported that STAT1 and STAT6 are expressed in synovial tissue of RA [42]. Therefore, the expression of p-JAK1, p-STAT1 and p-STAT6 in FLS was further investigated by using TNF-α-induced FLS as an in vitro cell model in our study. Our results showed that SKN could down-regulate phosphorylation of JAK1, STAT1 and STAT6 in TNF-α-induced FLS, suggesting that SKN could significantly inhibit the abnormal activation of the SOCS1/JAK/STAT signaling pathway in FLS.

## Conlusion

In conclusion, SKN significantly attenuated synovium abnormal proliferation of inflamed joints and reduced the serous secretion of IL-6, IL-1β and TNF-α in collagen-induced arthritis (CIA) mice. In addition, we demonstrated SKN suppressed the migration and invasion of TNF-α-induced FLS from rheumatoid arthritis patient. Furthermore, we found that SKN targeted SOCS1 and inhibited the phosphorylation of JAK1/STAT1/STAT6 in synovial tissues as well as FLS. Taken together, SKN represents a promising new anti-RA drug candidate that has anti-synovitis effect in CIA mice and inhibits TNF-α-induced FLS by targeting SOCS1/JAK/STAT signaling pathway. These findings demonstrate that SKN has anti-synovitis effect and has great potential to be a new drug for the treatment of rheumatoid arthritis.

## Supplementary Information

**Additional file 1**. SKN has no obvious hepatotoxicity and reproductive toxicity measured by HE.

**Additional file 2**. With the passage of time, the out-growth of FLS became more obvious.

**Additional file 3**. FLS was cultured with SKN and/or TNF-α for 24 h, then measured by MTT assay for cell cytotoxicity detection.

**Additional file 4**. Effect of shikonin(SKN) on the cell cycle of synovial fibroblasts.

**Additional file 5**. Effect of shikonin(SKN) on apoptosis of synovial fibroblasts.

## Data Availability

The datasets used or analysed during the current study are available from the corresponding author on reasonable request.

## Abbreviations

BSA: Bovine serum albumin
CIA: Collagen induced arthritis
DMSO: Dimethyl sulfoxide
FLS: Fibroblast like synoviocytes
FN: Fibronectin
IL-6: Interleukin-6
JAK/STAT: Janus kinase/signal transducer andactivator of transcription
MTX: Methotrexate
RA: Rheumatoid arthritis
SKN: Shikonin
SOCS: Suppressor of cytokine signaling
TGF-β: Transforming growth factor-β
