# Supplementary material for: Shikonin attenuates rheumatoid arthritis by targeting SOCS1/JAK/STAT signaling pathway of fibroblast like synoviocytes
Source: Chin Med. 2021 Oct 2;16:96. doi: 10.1186/s13020-021-00510-6 (PMC8487562; doi:10.1186/s13020-021-00510-6)
Supplement: Supplementary file 1 — Additional file 1. SKN has no obvious hepatotoxicity and reproductive toxicity measured by HE. [file 13020_2021_510_MOESM1_ESM.pptx]

## Slide 1
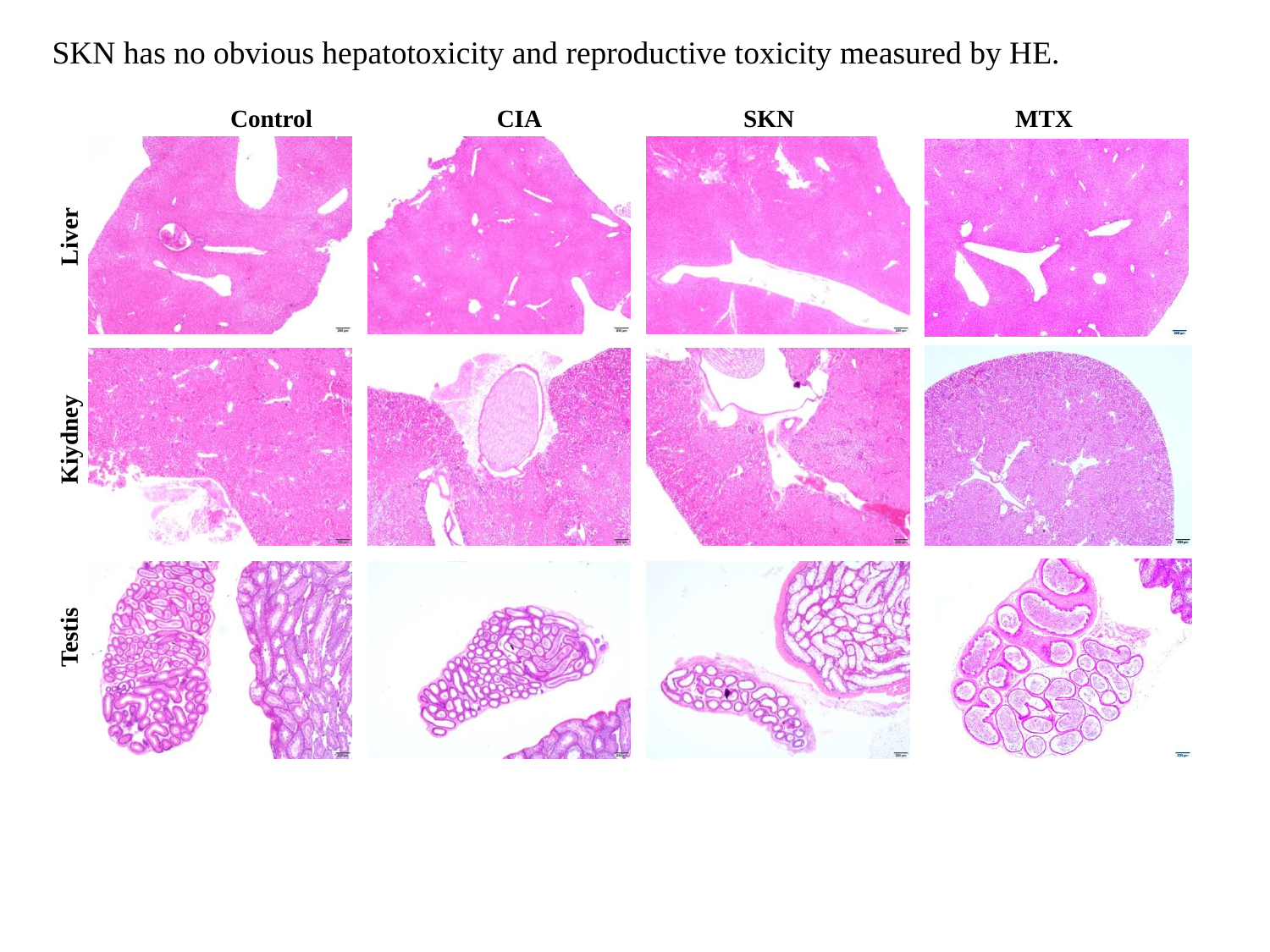

SKN has no obvious hepatotoxicity and reproductive toxicity measured by HE.
Control CIA SKN MTX
Testis Kiydney Liver
