# Supplementary figures and images for: Shikonin attenuates rheumatoid arthritis by targeting SOCS1/JAK/STAT signaling pathway of fibroblast like synoviocytes
Source: Chin Med. 2021 Oct 2;16:96. doi: 10.1186/s13020-021-00510-6 (PMC8487562; doi:10.1186/s13020-021-00510-6)

## Slide 1
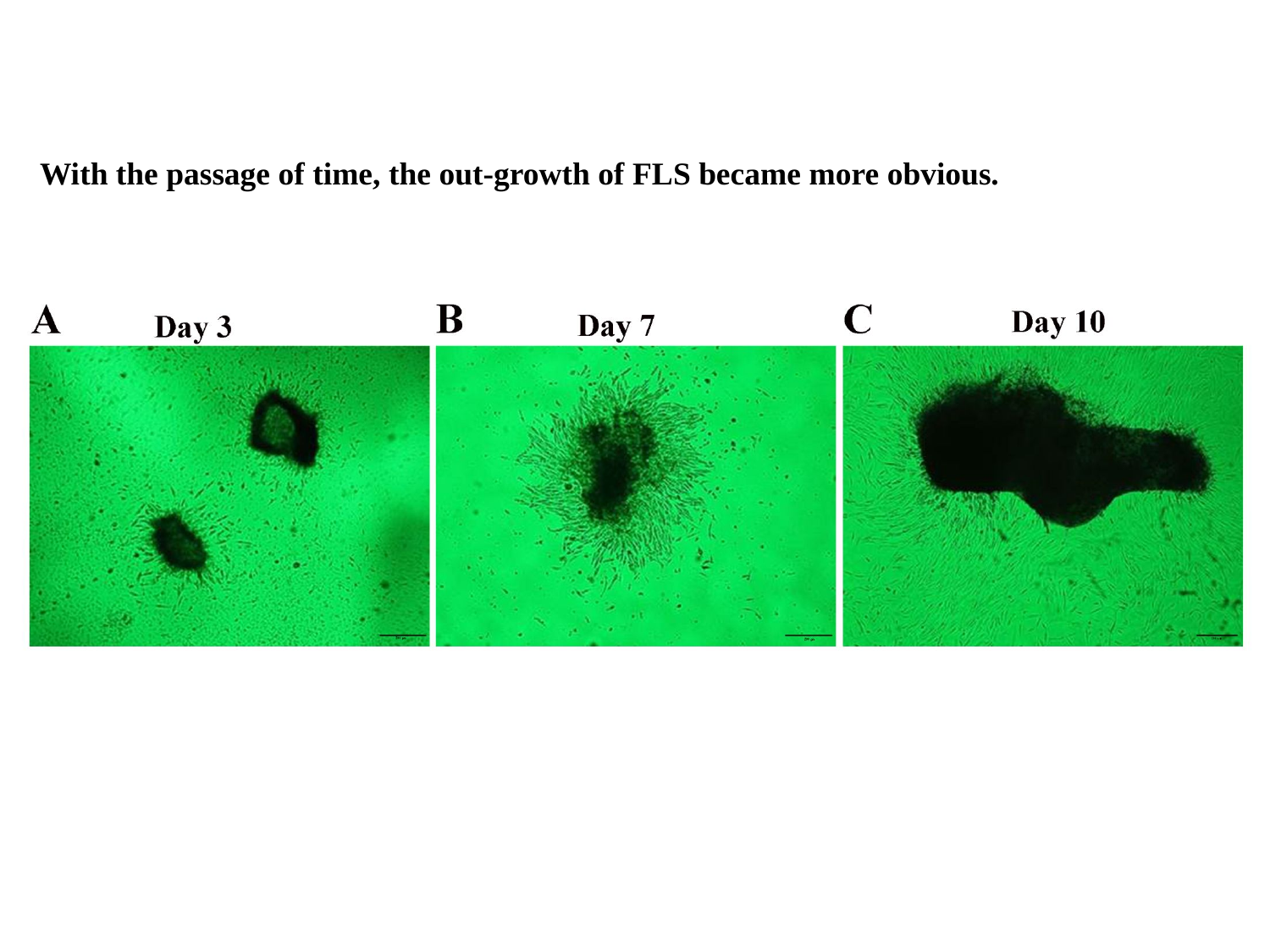

With the passage of time, the out-growth of FLS became more obvious.

Supplement: Supplementary file 2 — Additional file 2. With the passage of time, the out-growth of FLS became more obvious. [file 13020_2021_510_MOESM2_ESM.pptx]
