# Supplementary material for: Shikonin attenuates rheumatoid arthritis by targeting SOCS1/JAK/STAT signaling pathway of fibroblast like synoviocytes
Source: Chin Med. 2021 Oct 2;16:96. doi: 10.1186/s13020-021-00510-6 (PMC8487562; doi:10.1186/s13020-021-00510-6)
Supplement: Supplementary file 3 — Additional file 3. FLS was cultured with SKN and/or TNF-α for 24 h, then measured by MTT assay for cell cytotoxicity detection. [file 13020_2021_510_MOESM3_ESM.pptx]

## Slide 1
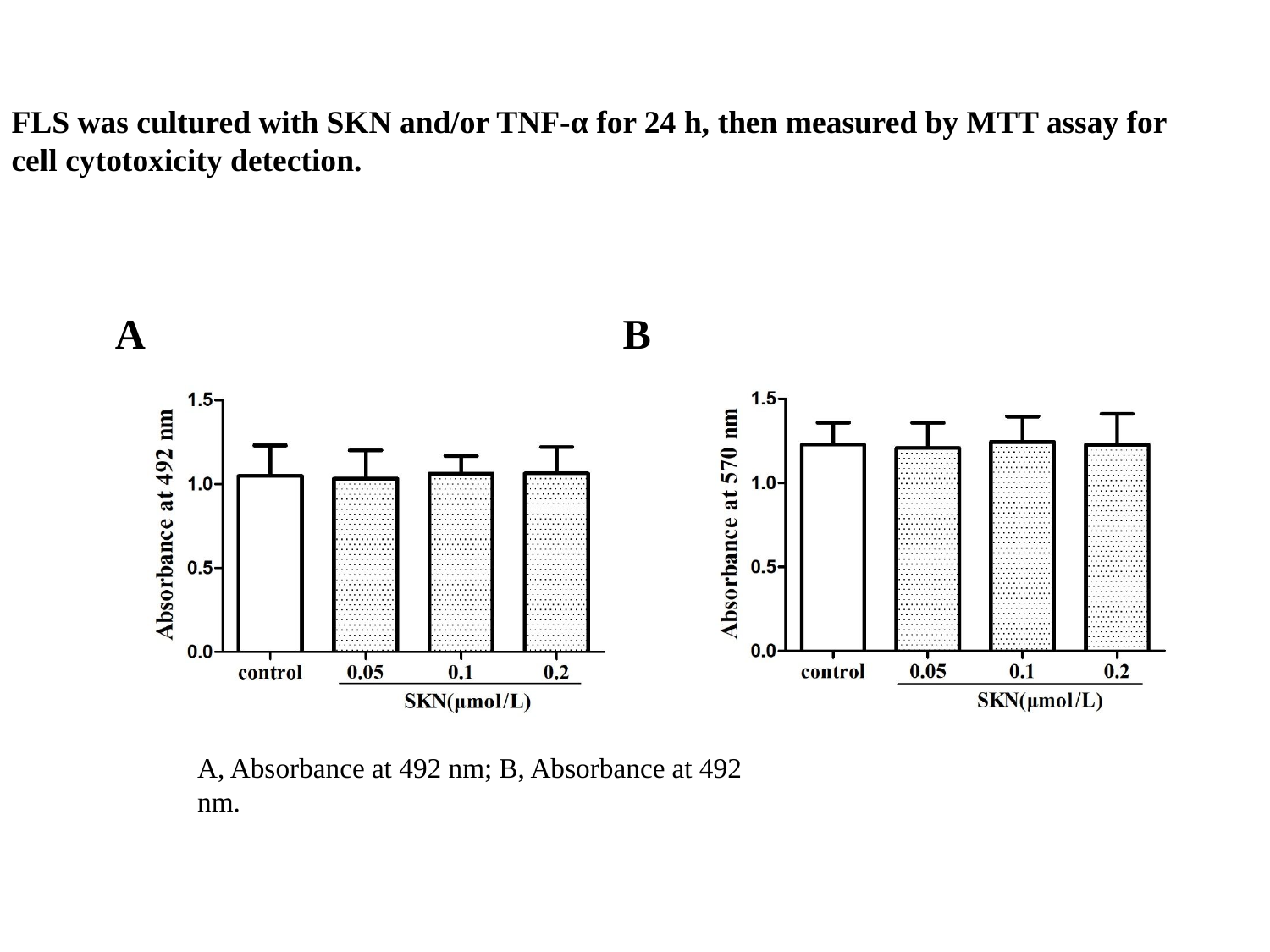

FLS was cultured with SKN and/or TNF-α for 24 h, then measured by MTT assay for cell cytotoxicity detection.
A
B
A, Absorbance at 492 nm; B, Absorbance at 492 nm.
