# Supplementary material for: Shikonin attenuates rheumatoid arthritis by targeting SOCS1/JAK/STAT signaling pathway of fibroblast like synoviocytes
Source: Chin Med. 2021 Oct 2;16:96. doi: 10.1186/s13020-021-00510-6 (PMC8487562; doi:10.1186/s13020-021-00510-6)
Supplement: Supplementary file 4 — Additional file 4. Effect of shikonin(SKN) on the cell cycle of synovial fibroblasts. [file 13020_2021_510_MOESM4_ESM.pptx]

## Slide 1
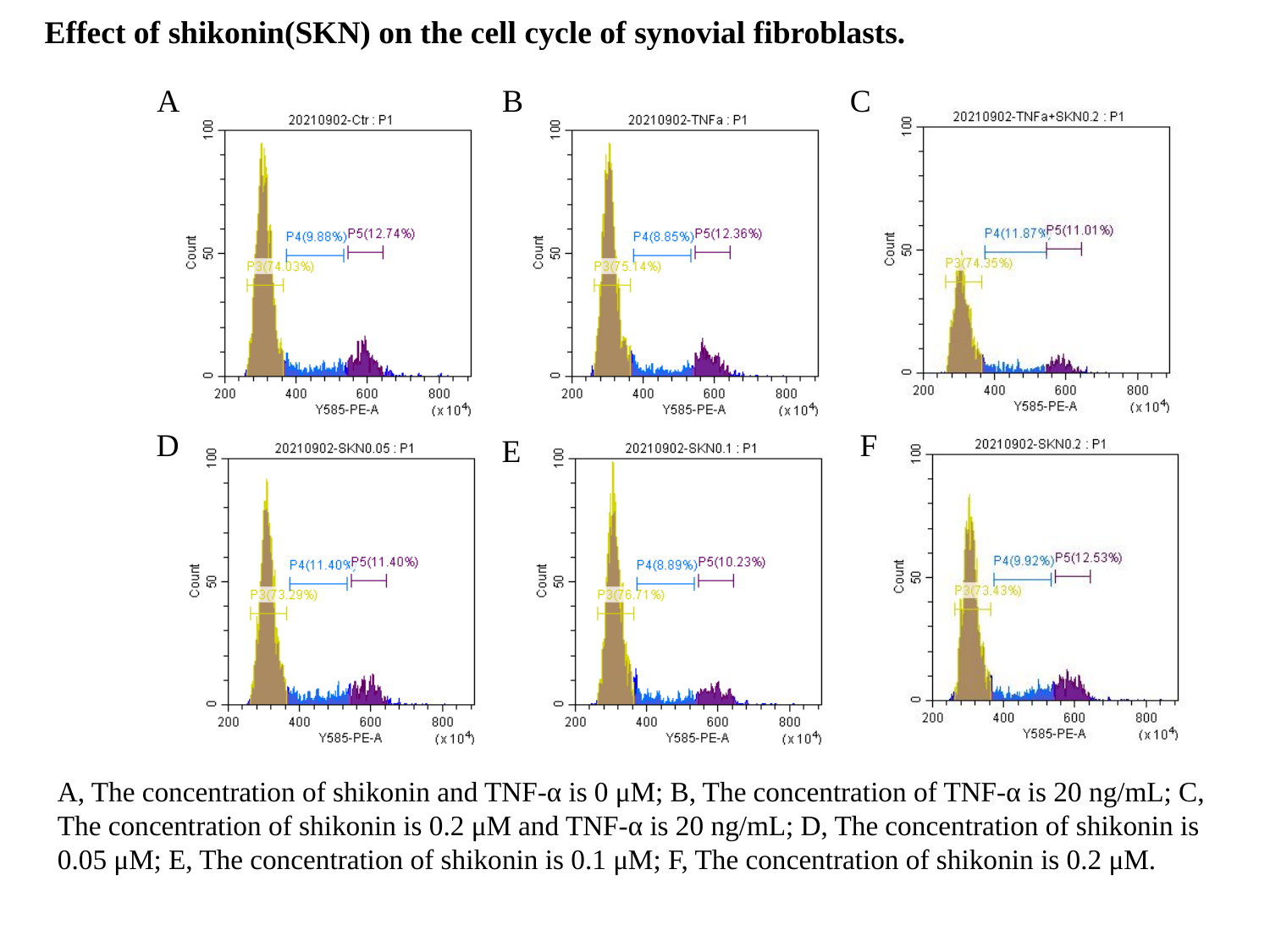

Effect of shikonin(SKN) on the cell cycle of synovial fibroblasts.
A
B
C
D
F
E
A, The concentration of shikonin and TNF-α is 0 μM; B, The concentration of TNF-α is 20 ng/mL; C, The concentration of shikonin is 0.2 μM and TNF-α is 20 ng/mL; D, The concentration of shikonin is 0.05 μM; E, The concentration of shikonin is 0.1 μM; F, The concentration of shikonin is 0.2 μM.
